# Witchcraft accusations against older women in Sub-Saharan Africa: a narrative review and community-based inclusive development framework synthesis

**DOI:** 10.3389/fpubh.2026.1861837

**Published:** 2026-07-22

**Authors:** Eva Yin-han Chung, Isaac Makponga

**Affiliations:** 1 Faculty of Medicine, Health and Life Science, Swansea University, Swansea, United Kingdom; 2 Worcestershire Acute Hospitals NHS Trust, Alexandra Hospital, Redditch, Worcester, United Kingdom

**Keywords:** community-based rehabilitation, older women, public health, social exclusion, witchcraft accusations

## Abstract

Witchcraft accusations against older women persist as a serious yet underexamined source of harm and exclusion in parts of sub-Saharan Africa, particularly in Ghana. While existing research has explored witchcraft beliefs and individual cases, comparatively less attention has been given to the longer-term health, social and participation impacts experienced by accused older women. This study addresses this gap through a two-stage qualitative evidence synthesis drawing on qualitative studies, ethnographies, policy documents, organizational reports, and human rights literature, primarily from Ghana and supported by comparative material from Nigeria and Malawi. Stage one employed a critical narrative review to synthesize fragmented evidence on the social, cultural and institutional processes shaping accusation and treatment. Stage two applied a framework synthesis, in which themes generated inductively were mapped onto the Community-Based Rehabilitation (CBR) Matrix to examine implications for participation and inclusion. The findings indicate that accusations disproportionately affect older women facing intersecting vulnerabilities related to gender, aging, poverty and social marginality, and are associated with violence, displacement, stigma, and prolonged exclusion from health services, livelihoods and community life. When interpreted through a community-based inclusive development lens, these patterns suggest constraints across health, education, livelihood, social and empowerment domains. The study highlights the potential importance of culturally informed, rights-oriented and participatory approaches that integrate advocacy, protection and rehabilitation, while strengthening institutional accountability and community-based support mechanisms. These findings are grounded in the contexts examined and offer implications for understanding and addressing witchcraft-related harm in similar settings.

## 1 Background

Witchcraft accusations remain a pervasive and deeply consequential social phenomenon across parts of sub-Saharan Africa (1). Although historically present across Europe, Asia and the Americas, contemporary accusations in many African contexts continue to shape community relations, social order and the lived experiences of vulnerable groups (2). Within these settings, witchcraft is often conceptualized not merely as a belief system, but as an interpretive framework through which misfortune, illness and interpersonal conflict are understood. This perception is underpinned by long-standing cultural traditions that embed witchcraft within wider systems of morality, cosmology and social regulation (3).

The figure of the “witch” is particularly gendered and age-specific. Older women, especially those who are widowed, socially marginalized or living in poverty, are disproportionately targeted (3). Anthropological work by scholars has shown that witchcraft beliefs frequently conflate age, gender and spiritual power, producing a narrative in which older women are perceived as potentially harmful, deviant or morally suspect (4). In many communities, physical signs of aging, disability or social withdrawal are reinterpreted as markers of supernatural danger. This dynamic contributes to processes of stigma, violence and social exclusion, particularly when accusations are acted upon, rather than arising from belief alone (5).

In Ghana, witchcraft continues to shape interpretations of misfortune and everyday social interactions. The persistence of witch camps, often described as spaces to which accused individuals are relocated for protection, illustrates the severity of community responses to allegations (6). These camps embody a dual reality: they protect women from immediate violence while simultaneously isolating them from family networks, livelihoods and political participation (7). Such practices highlight the intersection of gender, age, ethnicity, economics and customary law.

Despite growing international concern about the human rights abuses associated with witchcraft accusations, older women in this context, particularly in Ghana, remain largely overlooked in both academic research and state policy. Existing scholarship has predominantly focused on cultural explanations, anthropological analyses and historical narratives surrounding witchcraft (7–9), while far less attention has been directed toward its public health dimensions or the specific factors that position older women as particularly susceptible to accusation. There is also limited examination of how accused women are treated within their communities and how these experiences unfold over time. The social, psychological and economic impacts of accusation, including long-term exclusion, trauma and loss of livelihood, remain insufficiently explored. These gaps hinder a full understanding of how structural inequalities, community norms and local power dynamics sustain patterns of accusation and constrain opportunities for advocacy, protection and rehabilitation. From a community-based rehabilitation (CBR) perspective, such gaps also limit the development of evidence-informed strategies that promote equal participation and inclusion (10), making it difficult to design contextually grounded interventions that address the intersecting health, social and livelihood challenges faced by accused older women.

The aim of this study is to explore the social, cultural and economic factors contributing to the accusation of older women as witches in sub-Saharan African contexts, with a primary focus on Ghana and comparative insights from selected settings, and to examine the effects of these accusations on wellbeing, safety and social inclusion. The study further employs a community-based rehabilitation framework to consider how these dynamics shape opportunities for health, participation and inclusion.

### 1.1 Research questions

RQ1. What social, cultural and institutional factors contribute to witchcraft accusations against older women in sub-Saharan African contexts, particularly Ghana, and how do these dynamics shape their experiences, wellbeing and treatment within their communities?
RQ2. How are witchcraft accusations associated with the social, psychological and public health wellbeing of older women, and what forms of exclusion or harm arise from these experiences?
RQ3. What do these findings suggest for understanding constraints on health, participation and inclusion from a community-based inclusive development and rehabilitation perspective?

## 2 Methods

This study uses a two-stage qualitative evidence synthesis. Existing research provides only partial insight into the long term effects of witchcraft accusation, which indicates a need for approaches that can address the diversity and fragmentation of the available evidence. Although earlier scholarship has examined witchcraft as a cultural system or documented individual cases, much less attention has been given to the sustained and embodied consequences for older women who are targeted. This study responds by drawing together varied sources, including qualitative studies, ethnographies, organizational reports and community testimonies, to build a clearer understanding of these experiences.

Stage 1 employs a critical narrative literature review to address RQ1 and RQ2, and Stage 2 uses a framework synthesis informed by the CBR and CBID Matrix to address RQ3. This approach is appropriate because research on witchcraft accusations is heterogeneous, dispersed across disciplines, and embedded within highly localized contexts, whereas examining implications for participation and inclusion requires a structured, rights-based analytical framework (2). Qualitative evidence synthesis is well suited to this field because it supports the systematic integration of diverse qualitative materials and offers interpretative depth beyond individual studies (11). Framework synthesis further provides a systematic structure for organizing and interpreting diverse forms of evidence and is recognized as effective for addressing complex public health and policy questions (12). This combined approach offers a coherent strategy for addressing the study aim within a broad and fragmented evidence base.

### 2.1 Stage 1: critical narrative review (RQ1–RQ2)

A critical narrative review was selected because the evidence comprises ethnographies, qualitative studies, legal and policy texts, NGO reports and gray literature. Narrative review methods allow interpretive synthesis of such diverse sources and are appropriate where terminology and methodological standards vary considerably (13). A systematic review was not feasible and PRISMA reporting was not applied, as the aim was conceptual depth rather than protocol-driven aggregation.

The literature search was conducted across multiple databases, including AgeInfo, the international bibliography of the social sciences (IBSS), Web of Science Core Collection, Sociological Abstracts, and Social Services Abstracts. These searches were supplemented by the university library discovery system, hand-searching of reference lists, and snowballing techniques to identify additional relevant studies. Search terms combined keywords relating to the population (e.g., “older women”, “older adults”, “older adults”), the phenomenon of interest (e.g., “witchcraft”, “sorcery”, “occult”, “magic”), and the geographical context (e.g., “Africa”, “Sub-Saharan Africa”). Searches were limited to English-language publications between 1984 and 2026 to capture both historical and contemporary perspectives.

To enhance transparency, explicit inclusion and exclusion criteria were applied. Studies were included if they focused on older women, generally defined as aged 60 years and above, and addressed witchcraft or related concepts within African contexts, particularly Sub-Saharan Africa. Both qualitative and mixed-methods research were considered, alongside peer-reviewed studies, ethnographies, and organizational or policy reports. Studies were excluded where they were not published in English, were conducted outside African contexts, or did not directly engage with the intersection of older women and witchcraft. Additional exclusions were applied to sources lacking sufficient relevance to the research questions.

A structured screening process was undertaken. Across all databases, 8,444 records were initially identified. Following the removal of duplicates and preliminary filtering, 4,545 records were retained for further screening, with 3,899 duplicates excluded. Titles and abstracts were screened for relevance, followed by full-text assessment of eligible studies. Final inclusion was based on direct relevance to the research focus, specifically the intersection of older women, witchcraft accusations, and African contexts. Although a formal PRISMA framework was not applied due to the narrative design, the review followed a transparent and staged selection process to ensure methodological clarity.

Data extraction was conducted using a structured charting framework that captured definitions and conceptualisations of witchcraft, pathways to accusation, forms of treatment, and impacts on physical, social, economic, and psychological wellbeing. The synthesis involved iterative reading, thematic grouping, and reflexive memoing to support analytical rigor and consistency (14). This process enabled the identification of patterns across diverse sources while maintaining sensitivity to contextual variation.

Given the heterogeneity of the evidence base, a formal quality appraisal tool was not applied. Instead, studies were evaluated based on their thematic relevance, conceptual contribution, and credibility, including the use of recognized academic databases and established organizational sources. Particular emphasis was placed on the depth and richness of qualitative insight and the extent to which studies contributed to understanding the lived experiences of older women. This approach is consistent with critical narrative review methodologies, which prioritize interpretive depth and conceptual contribution over standardized methodological scoring. Evidence was therefore synthesized interpretively rather than weighted quantitatively.

Gray literature, including NGO reports, organizational publications, and human rights documentation, was included due to the limited representation of this topic within peer-reviewed academic literature. These sources provide important contextual and experiential insights that are often absent from formal research studies. However, such materials were used primarily for contextualization and triangulation and were not treated as equivalent to peer-reviewed empirical research.

### 2.2 Stage 2: framework synthesis (RQ3)

Findings from Stage 1 were subsequently analyzed through a framework synthesis (12) using the CBR Matrix to address RQ3. This approach integrates inductive insights from the narrative review with a deductive rights-based structure. The CBR Matrix (15) was selected for this stage of analysis. Its five domains of Health, Education, Livelihood, Social and Empowerment provide a coherent structure for interpreting how witchcraft accusations shape participation, inclusion and overall wellbeing. Grounded in community participation, stigma reduction and rights-based development, the CBR Matrix is well suited to examining community-driven processes such as witchcraft accusations. This synthesis enabled a structured assessment of how witchcraft accusations undermine equal participation and what this implies for community-based rehabilitation and inclusive development.

## 3 Results

### 3.1 Stage 1 critical narrative synthesis

This stage synthesizes evidence drawn from qualitative studies, ethnographies, NGO and policy reports, and human rights documentation concerning witchcraft accusations directed at older women in sub-Saharan Africa, with particular emphasis on Ghana and comparative material from Nigeria and Malawi. Despite variation in disciplinary orientation and methodological approach, the literature reveals consistent patterns relating to structural vulnerability, pathways to accusation, treatment following accusation, and the cumulative social, psychological, and public health consequences of exclusion and displacement.

#### 3.1.1 Structural vulnerability and disproportionate accusation

Across the reviewed literature, older women are consistently positioned as disproportionately vulnerable to witchcraft accusation due to the intersection of gender, aging, poverty, and socially authoritative belief systems. Evidence from Ghana demonstrates that accused women are commonly widows or socially marginalized older women characterized by low literacy, declining health, economic dependency, and limited family protection (16–18). Within these contexts, age-related bodily changes, reduced productivity, and social marginality are frequently interpreted as markers of occult threat, reflecting locally embedded cultural logics that construct vulnerability as suspicion (16).

These patterns are not confined to Ghana. Comparable evidence from Nigeria and Malawi suggests that such vulnerabilities are structurally reproduced across different settings. Among Igbo and Yoruba populations in Nigeria, as well as in parts of Malawi, visible signs of aging and socio-economic deprivation similarly intensify exposure to suspicion and accusation (17, 18). Across these contexts, accusations are embedded within broader social processes, including gendered expectations, inheritance disputes, and intergenerational tensions, particularly in environments characterized by economic uncertainty and social strain.

#### 3.1.2 Pathways to accusation and communal validation

The narrative synthesis indicates that accusations typically arise through socially patterned pathways linked to experiences of misfortune. In Ghanaian contexts, accusations frequently coalesce around illness, death, infertility, or crop failure, with witchcraft discourse functioning as a culturally authoritative explanatory system in contexts where biomedical access is limited and structural inequalities are pronounced (5, 8). Suspicion often originates within households or neighborhood networks and is subsequently validated through the involvement of religious leaders, spiritual intermediaries, or traditional authorities, transforming individual suspicion into collective judgement (16, 17).

Religious actors play a particularly influential role in Ghana, where prophets and pastors may identify older adult female relatives as sources of spiritual harm, thereby intensifying intra-family conflict and legitimizing exclusion (19, 20). Shrine custodians and traditional authorities further regulate access to protective or exclusionary spaces, reinforcing long-term marginalization.

Comparable pathways are evident in Nigeria and Malawi, where misfortune is similarly interpreted through spiritual frameworks and accusations are reinforced through communal validation. Although the specific mediating actors differ, the process of transforming suspicion into socially sanctioned judgement remains consistent and highlights the enduring authority of local cosmologies in shaping responses to adversity (7, 21).

#### 3.1.3 Treatment following accusation and institutional contexts

Following accusation, older women are typically subjected to severe forms of social sanction that unfold within family and community settings. In Ghana, accusations commonly originate within extended households and escalate to physical assault, public humiliation, exclusion, or forced relocation to long-established northern settlements such as Gambaga, Kukuo, Gnani, and Kpatinga. Conditions within these settlements are widely reported as inadequate, characterized by poor shelter, limited sanitation, constrained livelihoods, restricted mobility, and persistent stigma that obstructs return to home communities (16). Subsequent human rights investigations confirm enduring deficits in food security, safe housing, and access to health care, producing prolonged precarity.

In other sub-Saharan African contexts, including Nigeria and Malawi, the literature similarly documents patterns of physical abuse, expulsion from households, and community-level punishment following accusation (6, 22, 23). While the forms and organization of responses vary across settings, these accounts consistently highlight the central role of family and community structures in enforcing accusation and exclusion, often resulting in displacement, social isolation, and loss of protection.

The literature further documents extreme outcomes that reveal the lethal potential of unchecked accusation. National and regional evidence describes cases of lynching and grannicide, in which grandsons killed grandmothers following allegations involving destiny theft, spiritually induced illness, or recurrent dreams. The killing of Akua Denteh in 2020 is frequently cited as emblematic of such outcomes and of broader failures of protection (1, 19).

These practices persist within contexts of significant institutional fragility. Legal and policy gaps, inconsistent law enforcement, and limited state capacity have been repeatedly identified as enabling factors (16). Reliance on externally driven, rights-based interventions often privileges Eurocentric norms over indigenous epistemologies, exacerbating tensions between global human rights standards and local belief systems. The literature highlights a persistent dilemma: engagement with local ontologies risks legitimizing harmful practices, while imposition of universal norms may provoke resistance and inadvertently heighten risk for accused women. Institutional weakness therefore constrains practitioners' capacity to intervene and permits the continuation of exclusionary practices (24).

#### 3.1.4 Psychological, social, and public health consequences

The critical narrative synthesis identifies extensive and interrelated consequences of accusation across psychological, social, and public health domains. Empirical studies conducted in Ghanaian settlements report elevated prevalence of depression and anxiety, with some indicating that more than half of residents are affected (25). Trauma is linked not only to the act of accusation itself but also to displacement, public shaming, violence, and abandonment by family members (5). Persistent fear, disrupted sleep, and chronic insecurity are widely reported, alongside symptoms consistent with post-traumatic stress following physical or verbal assault (7, 26).

Stigma operates as a chronic psychological stressor that sustains long-term harm. Once labeled a witch, the identity becomes socially entrenched and resistant to reversal, contributing to diminished self-esteem and erosion of personal agency (3). Opportunities for rehabilitation or reintegration are severely constrained, while mental health services within camps and surrounding communities remain scarce, allowing psychological distress to remain untreated and frequently become chronic (27–29).

Social consequences of accusation include abrupt displacement and the systematic erosion of social belonging. Accused individuals are frequently compelled to abandon their homes and relocate to isolated settlements, resulting in immediate loss of kinship ties, social identity, and community support (20, 25). Stigma associated with witchcraft accusations also reshapes wider social dynamics. Quantitative evidence suggests that communities with high levels of belief in witchcraft exhibit lower interpersonal trust, including diminished trust in relatives and neighbors, signaling weakened in-group cohesion (30, 31). Families may sever ties with accused relatives to protect their own reputations, intensifying social isolation.

Public health impacts are severe, cumulative, and structurally embedded. Settlement environments commonly lack clean water, adequate sanitation, and safe housing, exposing residents to infectious disease and environmental risk (28, 29). Food insecurity is widespread, with malnutrition particularly pronounced among older women (26). Access to healthcare is substantially constrained; residents frequently experience untreated chronic conditions such as hypertension, arthritis, and vision impairment due to financial barriers, geographic isolation, or exclusion from national health insurance schemes (25, 32). Injuries sustained during assaults or attempted killings often remain untreated, leading to long-term disability or preventable mortality (33). These factors interact to reinforce a cycle in which poor health, poverty, and social exclusion mutually exacerbate one another (5).

Comparable patterns are evident across Nigeria and Malawi, where displacement, stigma, and social fragmentation similarly shape lived experiences. Although less concentrated within camp systems, these contexts exhibit reduced interpersonal trust and weakened social cohesion in communities where witchcraft beliefs are prevalent (31, 34). Across all settings, structural inequalities and limited access to services compound the long-term impacts of accusation.

#### 3.1.5 Summary of stage 1 findings

Overall, the synthesis demonstrates that witchcraft accusations against older women represent systemic and cumulative social processes rather than isolated incidents. Evidence from Ghana illustrates how these dynamics can become institutionalized through structured displacement practices, while comparative insights from Nigeria and Malawi confirm that similar processes are reproduced across diverse contexts. Structural vulnerability shaped by gender, aging, economic precarity, and intergenerational tensions renders older women particularly susceptible to accusation, while culturally authoritative belief systems legitimize suspicion (6, 7). Institutional fragility further constrains effective protection, allowing violence, exclusion, and displacement to persist. Across sub-Saharan Africa, these processes generate sustained psychological, social, and public health harms that remain insufficiently addressed within existing legal and policy frameworks (9, 22, 35).

### 3.2 Stage 2 framework synthesis using the CBR matrix

This stage analyses findings from the Stage 1 critical narrative review through a framework synthesis informed by the Community Based Rehabilitation CBR Matrix (15), in order to examine how witchcraft accusations directed at older women constrain participation, inclusion, and wellbeing. Building on the comparative synthesis in Stage 1, the analysis situates these implications within a broader sub-Saharan African context. The findings indicate that witchcraft accusations directed at older women are associated with constraints across multiple dimensions of wellbeing and participation (16, 17, 22). In this stage, themes identified inductively in Stage 1 (e.g., displacement, stigma, restricted livelihoods, and barriers to healthcare) were subsequently mapped onto the five domains of the CBR Matrix. This mapping was interpretive and iterative, allowing themes to be aligned with domains based on their primary relevance rather than being coded directly into predefined categories. When interpreted through the community-based rehabilitation (CBR) Matrix, these patterns point to constraints that may affect health, social inclusion and opportunities for engagement within community life. The following sections outline these implications within the five CBR domains, highlighting areas where community-based inclusive development processes may face particular limitations.

#### 3.2.1 Health domain

Across the cases examined, aging-related physical and cognitive changes are sometimes interpreted within communities as indicators of supernatural involvement (16). Such interpretations may influence how illness is understood and managed, potentially reducing reliance on biomedical services and delaying access to appropriate care. For individuals relocated to designated settlements known as witch sanctuaries or camps, reported conditions such as inadequate housing, limited sanitation and restricted healthcare access contribute to environments associated with ongoing health risks (6, 16). Themes relating to untreated illness, delayed care-seeking, and exposure to poor living conditions were therefore mapped to the health domain, reflecting constraints on access to services and health promotion.

From a CBR perspective, these patterns suggest potential challenges for health promotion, preventive care and basic rehabilitation at community level, particularly where spiritual explanatory frameworks shape help-seeking behaviors (26). Strengthening accessible and culturally informed health education could therefore play a role in supporting community-based inclusive responses (36). Limited confidence in biomedical services also affects the use of medical care, as individuals may delay or avoid clinical treatment in favor of spiritual interventions (21). Improving the availability and acceptability of primary healthcare within communities can help encourage earlier engagement with formal services and reduce avoidable health complications.

#### 3.2.2 Education domain

The evidence indicates that many accused women have limited or no formal education, which restricts their ability to navigate legal processes, access social services or participate meaningfully in community awareness programmes, (16). At the broader community level, the lack of structured learning about aging, disability or mental health reinforces supernatural interpretations of misfortune, thereby sustaining the conditions under which accusations occur (24). These patterns, initially identified as themes relating to knowledge gaps, belief reinforcement, and limited health literacy in Stage 1, were mapped to the education domain.

Education is a central tool for reducing witchcraft accusations and related violence, as it fosters critical thinking, rationality and scientific temper, enabling individuals and communities to question superstitious beliefs and reject supernatural explanations for misfortune (37). Education also strengthens health literacy by helping people understand natural causes of illness and discouraging reliance on witchdoctors for health related problems, which can reduce scapegoating and the construction of alleged witches (38). Furthermore, improving access to education enhances economic opportunities and reduces poverty related vulnerabilities that often fuel accusations, while also cultivating critical consciousness that encourages communities to question entrenched institutional practices, challenge the authority of witchdoctors and dismantle cultural norms that legitimize witch hunting (37). Both formal and non formal approaches, including community awareness activities, school based learning and participatory critical pedagogy, are identified as essential for long term change (5, 37).

#### 3.2.3 Livelihood domain

Several studies describe a relationship between economic insecurity and the likelihood of being accused, particularly among widows and older women lacking stable income or family support (17, 18). Following relocation, reported restrictions on mobility and limited access to land or economic activities may reduce opportunities for self-sufficiency (16). Themes relating to economic dependency, loss of income, and restricted mobility were mapped to the livelihood domain. Within a CBR framework, such patterns suggest constraints on livelihood development, skills acquisition and access to social protection (15). Addressing these limitations may require community-level strategies that support income generation, economic participation and social safety nets for highly vulnerable groups.

#### 3.2.4 Social domain

Social exclusion appears frequently in the available evidence. Accusations are often described as originating within extended family settings and escalating through involvement of local authority figures, contributing to strained family relationships and, in some cases, displacement (19, 20). Conditions in settlement camps, including reduced mobility and persistent stigma, may limit opportunities for social participation (16). Weak enforcement of legal protections has also been noted in several studies (24). These themes, particularly stigma, displacement, and breakdown of social relations, were mapped to the social domain, reflecting constraints on participation and inclusion. Within the CBR social domain, these findings highlight potential barriers to community participation, family support, justice and social integration. Interventions aimed at strengthening community dialogue, legal literacy and participatory mechanisms may therefore support more inclusive local environments.

#### 3.2.5 Empowerment domain

The evidence suggests that individuals accused of witchcraft may have limited opportunities to participate in community decision-making or to influence processes affecting their wellbeing. Local human rights practitioners report challenges navigating tensions between community belief systems and international rights frameworks, which may restrict the scope for empowerment-focused interventions (24). The influential role of traditional and religious authorities, who may validate rather than contest accusations, can further reduce affected women's agency (19, 20). The absence of structured self-help groups or advocacy networks within the camps indicates limited avenues for collective organization (6, 7). Themes relating to reduced agency, lack of voice, and limited participation in decision-making processes were therefore mapped to the empowerment domain. From a CBR perspective, these observations point to constraints on community mobilization, leadership opportunities and the development of supportive peer networks.

## 4 Discussion

The findings highlight the multidimensional conditions shaping the experiences of older women accused of witchcraft across Ghana, Nigeria and Malawi. When interpreted through a community-based inclusive development (CBID) lens, these patterns point to structural and relational processes that influence advocacy efforts, protective mechanisms, rehabilitation pathways and the formulation of rights-based interventions. This discussion draws on the five CBR domains to examine how community-level and system-level responses may be strengthened, while remaining sensitive to the cultural environments in which accusations occur.

### 4.1 Advocacy within community-based inclusive development frameworks

From a community-based inclusive development perspective, advocacy in support of people accused of witchcraft may be strengthened through approaches that emphasize engagement with existing community structures, recognition of witchcraft as a lived social reality without legitimizing violence, and prioritization of safety, agency and choice. Dialogic and collaborative approaches such as community mediation, negotiated reintegration and protective monitoring have been identified in the literature as potentially supporting incremental shifts in attitudes toward aging, illness and dependency, while maintaining local legitimacy and reducing the risk of further harm (23, 35). Within this framework, advocacy is understood not as a purely normative or enforcement-driven intervention, but as a relational and structurally oriented practice shaped by the interaction between local belief systems, experiences of spiritual insecurity and external human rights frameworks.

Barriers to protection are frequently embedded within familial, communal and institutional dynamics. Findings from this study indicate that older women often encounter constraints arising from the familial origins of accusations, the mediating role of community intermediaries, and the limited enforcement or accessibility of legal safeguards (5, 7, 16). These conditions restrict the availability of formal safeguarding pathways, particularly where accusations are addressed within households or informal community processes. In many settings, traditional and religious authorities hold significant interpretive power over misfortune, illness and aging, and their influence may reinforce accusations where advocacy efforts are insufficiently embedded in local realities or where institutional accountability is weak (2, 19).

Evidence suggests that harm arises less from belief itself than from the social responses triggered by accusation. Schnoebelen (23) emphasizes that belief in witchcraft does not constitute the primary protection concern. Rather, risk emerges from actions taken in response to accusations, including violence, forced displacement, confinement and other serious human rights violations. Roxburgh (35) similarly demonstrates that externally driven anti-witchcraft campaigns in Ghana often frame witchcraft as irrational belief and witch camps as inherently abusive, without sufficiently recognizing the complex reality that, despite their harmful and exclusionary conditions, some accused individuals seek refuge in such spaces to avoid immediate violent reprisals where effective community or state protection is unavailable. This constitutes a protection dilemma in which exclusionary practices may persist not because they are protective in themselves, but because alternative safety mechanisms remain structurally inaccessible. Rights-based approaches that fail to acknowledge this reality may risk marginalizing local voices and undermining fragile protection mechanisms, particularly where fear, insecurity and weak state presence prevail (23).

From a CBID standpoint, this dilemma underscores the importance of dialogue and collective reflection as mechanisms for structural change, which may enable communities and institutions to reconsider how vulnerability, aging and dependency are addressed. Strengthening protection therefore may require attention to community-based mechanisms for early identification of risk, improved legal literacy, and stronger linkages between formal authorities and community structures. Social protection policies that address economic insecurity may further contribute to reducing precarity and strengthening household resilience (17, 18). Community-based mediation structures, supported by local governance actors, may provide an additional layer of protection by addressing disputes before escalation.

### 4.2 Community-based interventions and rehabilitation

Rehabilitation following witchcraft accusations is strongly shaped by displacement contexts and the conditions within designated settlements, where limited access to healthcare, sanitation, social participation and livelihood opportunities is frequently documented (16). These constraints affect both physical and psychosocial recovery and may undermine the feasibility and sustainability of community reintegration. From a community-based inclusive development perspective, rehabilitation extends beyond immediate safety and care to emphasize empowerment through the restoration of livelihoods, rebuilding of social relationships and support for aging related needs (10). Community-based counseling, facilitated family dialogue and participatory reintegration planning are suggested in the literature as potential approaches that may support older women in reclaiming social roles and navigating returns to home environments, particularly where stigma, fear or unresolved conflict persists. Locally developed reintegration protocols may also contribute to addressing relational harms arising during accusation processes.

Rehabilitation within a CBID framework is closely linked to broader community development and protection systems. The development of rights-based interventions is shaped by the cultural and social contexts in which witchcraft accusations occur, where locally embedded interpretations of misfortune may complicate direct implementation of universal human rights norms (1). These findings indicate that community-based programmes may need to move beyond nominal participation by enabling communities to influence decisions that affect their lives. Practitioners and policymakers may consider investing in institutional frameworks that support inclusive participation, integrate local knowledge with technical expertise, and embed participatory approaches into planning processes (39). By operationalising participation in this way, programmes can foster shared responsibility for safeguarding and reintegration and improve the long-term sustainability and effectiveness of community-based initiatives.

Participatory Planning and Review, together with participatory evaluation, can provide a practical mechanism through which principles of empowerment, shared responsibility and accountability are embedded within community-based rehabilitation responses to witchcraft accusations (40). Through structured collective reflection on risk, aging, vulnerability and social obligation, supported by dialogue with traditional and religious authorities, communities are able to identify locally appropriate responses and strengthen protective norms grounded in dignity, non-violence and equity. Participatory evaluation reinforces these processes by combining quantitative indicators of rehabilitation with qualitative and participatory methods (41), including storytelling and reflective dialogue, which enable accused older women to articulate their own experiences of recovery and reintegration. Simultaneously, these approaches support collective reflection on evolving beliefs related to illness, misfortune and aging, ensuring that rehabilitation interventions remain contextually grounded, responsive and aligned with locally defined measures of social transformation.

### 4.3 Implications for international communities

International actors therefore occupy a distinct enabling role, not as primary drivers of intervention design, but as contributors to the policy, funding, and normative conditions that allow locally grounded responses to be sustained for affected individuals and their families. This role includes providing flexible, longer-term funding, supporting capacity strengthening across health, social protection, and justice systems, and recognizing safeguarding approaches that are locally negotiated while remaining legible within international standards (1, 2, 33). Witchcraft accusation and related persecution constitute a severe and ongoing form of violence that disproportionately affects women, children, older persons, people with disabilities, and other socially marginalized groups, yet remains under-reported, weakly prosecuted, and inconsistently addressed within domestic and international legal frameworks (42, 43). Although freedom of belief is protected under international human rights law, the practices of accusation, torture, displacement, and killing associated with witchcraft clearly violate fundamental rights to life, dignity, and freedom from inhuman treatment, exposing persistent gaps between normative commitments and enforcement in practice (42, 43). At the same time, evidence indicates that external interventions focused narrowly on suppressing beliefs or criminalizing accusations may have unintended consequences, particularly where such beliefs function as informal mechanisms of order in contexts of institutional weakness. Framing witchcraft-related harm within intersecting global agendas on gender, aging, disability and development may help to elevate the issue in ways that remain contextually sensitive, allowing international engagement to support, rather than replace, community-based responses (34, 42).

## 5 Conclusion

This study demonstrates that witchcraft accusations directed at older women constitute a systemic and multidimensional form of exclusion that operates at the intersection of gender, aging, poverty, belief systems, and institutional fragility. Drawing on a two-stage qualitative evidence synthesis, grounded primarily in evidence from Ghana and supported by comparative material from other sub-Saharan African contexts, the findings indicate that accusations are not isolated cultural practices or solely belief-driven phenomena, but socially organized processes that produce sustained harm through violence, displacement, stigma, and loss of participation across health, education, livelihood, social, and empowerment domains. Interpreted through a community-based inclusive development perspective, these patterns reveal significant constraints on advocacy, protection, rehabilitation, and rights realization, particularly where community level responses are shaped by fear, spiritual insecurity, and weak state presence. The analysis underscores that effective responses must move beyond belief suppression or purely legalistic interventions to engage with the relational and structural conditions that sustain accusation and exclusion. Culturally informed, rights oriented, and participatory approaches that strengthen community dialogue, social protection, rehabilitation pathways, and institutional accountability are therefore essential. By situating witchcraft accusation within inclusive development and rehabilitation frameworks, this study contributes a structured and contextually grounded basis for advancing more legitimate, protective, and sustainable responses for older women at risk of accusation.
